# A novel trehalosamine isolated from *Bacillus amyloliquefaciens* and its antibacterial activities

**DOI:** 10.1186/s13568-019-0943-x

**Published:** 2020-01-14

**Authors:** Ying Wang, Bo Zhao, Yaping Liu, Linjing Mao, Xuanming Zhang, Wu Meng, Kechun Liu, Jie Chu

**Affiliations:** 1 Biology Institute, Qilu University of Technology (Shandong Academy of Sciences), 28789 East Jingshi Road Ji’nan, Shandong, 250103 China; 2 School of Medical Instrument and Food Engineering, University of Shanghai for Science and Technology, Shanghai, 200093 China; 3 College of Animal Science and Technology, Shandong Agricultural University, Tai’an,, 271018 Shandong China; 4 State Key Laboratory of Biobased Material and Green Papermaking (LBMP), Qilu University of Technology (Shandong Academy of Sciences), Jinan, Shandong 250353 China; 5 Key Laboratory of Shandong Microbial Engineering, College of Bioengineering, QiLu University of Technology (Shandong Academy of Sciences), Jinan, Shandong 250353 China

**Keywords:** Antibacterial activity, *Bacillus amyloliquefaciens*, Identification, Environmental friendly

## Abstract

*Bacillus amyloliquefaciens* has been widely used as a probiotic in the field of biological control,and its antibacterial compounds plays an important role in the prevention and control of plant, livestock and poultry diseases. It has the advantages of green, safe and efficiency. This study aims to separate and purify active ingredient from *Bacillus amyloliquefaciens* GN59 and study its antibacterial activity. A novel compound was isolated from GN59 by column chromatography on silica gel and HPLC purification. The chemical structure was identified as α-d-glucopyranosyl-(1 → 1′)-3′-amino-3′-deoxy-β-d-glucopyranoside (a,β-3-trehalosamine) on the basis of spectroscopic analysis. This is the first report about a,β-3-trehalosamine isolated from biological resources on an antibiotic activity against pathogenic bacterium. The 3′-neotrehalosamine displayed antibacterial activity across a broad spectrum of microorganisms, including different gram-positive and gram-negative bacteria, with minimal inhibitory concentration (MIC) values ranging from 0.5 to 0.7 mg/mL. The results indicated that the 3′-neotrehalosamine from GN59 might be a potential candidate for bactericide.

## Introduction

At present, more and more antibiotics have been discovered since Fleming invented penicillin in 1942 that saves countless people infected by microorganism (Mohr 2016; Lambrichts et al. 2017). However, along with the introduction of antibiotics into clinical practice, resistance by pathogenic bacteria has become one of the most important healthy point. More seriously, super-resistant bacteria against all antibiotics have been found such as methicillin-resistant *Staphylococcus aureus* (Kopp et al. 2004) and *NDM*-*1 bacteria* (Kumarasamy et al. 2010). Series of security issues including drug resistance, residual toxicity caused by antibiotics abuse have attracted more and more attention. It has become a valuable research hotspot to find a new and safe substitute with the urgent requirements of environmental protection, green and sustainable development.

In recent years, much attention has been drawn to natural antimicrobial substances synthesized by some microorganisms as antibiotics alternatives (Pellerito et al. 2018). *Bacillus* spp. have a wide distribution in the nature, and have been widely used as a probiotic in the field of biological control (Jager et al. 2018; Jezewska-Frąckowiak et al. 2018; Lefevre et al. 2017). Its antibacterial compounds play an important role in the prevention and control of plant, livestock and poultry diseases.

*Bacillus. amyloliquefacien* is a non-pathogenic aerobic gram-positive bacterium. It widely distributes in plant surface, soil, and air. During the metabolic process, it produces a mass of bioactive substances which have antibacterial activity, immunocompetence, antioxidant activity (Xiaolong et al. 2019). It also produces many kinds of enzymes such as alpha-amylase and protease, and shows high adaptability and stress resistance (Du et al. 2018). So it is generally applied in animal husbandry and aquaculture (Cawoy et al. 2014).

A number of *Bacillus* species play vital roles in controlling diseases due to their secondary antimicrobial metabolites, such as lipopeptide, macrolide and other active proteins (Chen et al. 2019; Ruan et al. 2019; Wang et al. 2019). Shashidar et al. (2017) have used antagonistic lipopeptides separated from *Bacillus* UCMB5113 to counteract pathogens. Pengfei et al. (2018) have reported a new antifungal compound, cyclic lipopeptide cyclic lipopeptide was isolated from *B. amyloliquefaciens* HAB-2. Abdallah et al. (2018) have indicated that lipopeptides from *B. amyloliquefaciens* strain 32a could act as promising biocontrol compounds to reduce the plant pathogen *Agrobacterium tumefaciens*.

In the present study, we isolated a novel bactericidal compound from the fermentation liquid of *B. amyloliquefaciens* GN59 using column chromatography as isolation and separation technique. Its chemical structure was identified as α-d-glucopyranosyl-(1 → 1′)-3′-amino-3′-deoxy-β-d-glucopyranoside (a,β-3-trehalosamine) on the basis of spectroscopic analysis. This is the first isolated a,β-3-trehalosamine that has antibacterial activity from biological resources. Further, we characterized the antimicrobial activity properties of a,β-3-trehalosamine. The minimal inhibitory concentration (MIC) to eight selected strains were between 0.5 and 0.7 mg/mL.

## Materials and methods

### Microorganisms

Different pathogenic bacterial strains were used to test the antimicrobial activity of metabolites, including *Staphylococcus aureus* ATCC6538, *Micrococcus luteus* CMCC280010, *Escherichia coli* CMCC44102, *Salmonella pullorum* CVCC533, C79-13, *Salmonella gallinarum* CVCC79201, C79-1, *Salmonella gallinarum* CVCC79207, C79-7, *Pasteurella multocida* CVCC474, C48-7 and *Salmonella enteritidis* ATCC13076. The strain of *B. amyloliquefaciens* was got from China General Microbiological Culture Collection Center. It was numbered as CGMCC 1.936, and named GN59 in this paper (http://www.cgmcc.net/index.html). GN59 was cultured in specific fermentation medium (30 g glucose, 7.0 g K_2_HPO_4_·3H_2_O, 3.0 g KH_2_PO_4_, 1.5 g (NH_4_)_2_SO_4_, 0.5 g trisodium citrate, 0.1 g MgSO_4_·7H_2_O in 1 L distilled water, pH 7.0) and the media was autoclaved at 121 °C for 20 min. Liquid cultures were shaken at 180 rpm at 37 °C for 42 h. All other strains were grown on lysogeny broth agar (LB) plates at 28 °C and cultured at 37 °C in LB medium.

### Chemicals and reagents

All the chemicals and reagents were analytical grade or highest available purity, and purchased from Sigma Chemical Co.

### Antimicrobial activity assay

The agar-well diffusion method according to the National Committee for Clinical Laboratory Standards (NCCLS) was used to measured the antimicrobial activity of each fraction (Salazar et al. 2017). Different pathogens were cultured in 50 mL of LB medium in 250 mL Erlenmeyer flasks and incubated at 37 °C with 180 rpm shaking for 12 h. LB agar plates were evenly covered with 1 × 10^7^ CFU/mL of suspensions in 100 μL and wells of 2.5-mm diameter were formed by a sterile cork borer. 20 μL of the separated fractions were added into the wells. Specific solvents were the negative controls and the antibiotic ampicillin (15 mg/mL) for gram-negative bacteria and vancomycin (50 mg/mL) for gram-positive bacteria were used as the positive controls. The plates were incubated at 37 °C for 12 h and the activities were determined by measuring the diameter of the inhibition zone. The experiments were performed in triplicate.

### Extraction, isolation and purification of active compound

The bacterial precipitate was removed by centrifugation at 8000*g* for 15 min, and the clear supernatant (2 L) was extracted with methanol (1:1, v/v) for three times (each time for 12 h) at room temperature to extract the active constituent. The combined solution was concentrated by rotary evaporator at 45 °C. Then the subfractions BA1–BA5 were isolated using silica column chromatography with petroleum ether/ethyl acetate (100:0, 75:25, 50:50, 25:75, 0:100, v/v) and the subfractions BA6–BA9 were isolated with ethyl acetate/methanol (75:25, 50:50, 25:75, 0:100, v/v). The active fraction BA8 was further purified by semi-preparative High Performance Liquid Chromatography (semi-HPLC, LC-20A, Shimadzu, Japan) using a YMC-Pack ODS-A column (250*20.0 mm) at a flow rate of 2.0 mL/min at 254 nm. Methanol (Sigma, USA) and H_2_O (7:3, v/v) were used as the mobile phase in a isocratic elution mode. All peak fractions were collected individually and concentrated via lyophilization to get six components, a (0.8 mg; tR 0.0–1.0 min), b (1.5 mg; tR 1.0–5.1 min) and c (63.1 mg; tR 5.1–5.5 min), d (64.1 mg; tR 5.5–6.5 min), e (1.1 mg; tR 6.5–7 min). Component c, d and e were found to contain glycoside components by TLC analyses (data are not shown). Then their antibacterial activity was detected to make sure the active constituent.

### The structure analysis of compound with antibacterial activity

The active compound of fermentation supernatant of GN59 was subjected to NMR and LC–MS analysis to obtain the chromatogram and the prospective mass spectra of the separated compound. NMR spectra was recorded (^1^H NMR 400 MHz and ^13^C NMR 100 MHz) on Bruker AMX400MHz Spectrometer (Bruker BioSpin GmbH, Ettlingen, Germany) using deuterated chloroform (CDCl_3_) as solvent. Mass spectrum was carried out using Varian 1200 L Mass Spectrometer (Varian India Pvt. Ltd., Powai, Mumbai).

### MIC and MBC of antimicrobial compound

The minimum inhibitory concentration (MIC) and minimum bactericidal concentration (MBC) of the antimicrobial compound from GN59 against eight tested pathogenic bacterium were determined by micro-broth dilution (Hoelzer et al. 2011). The antimicrobial compound was diluted in 96-well plates by serial dilutions using methanol. Then different bacterium of approximately 10^5^–10^6^ CFU/mL was added into each well, mixed gently and then incubated at 37 °C for 18–24 h. The MICs were recorded by comparing to the growth control and the last concentration. 10 μl of each mix liquids diluting with PBS were dropped onto Ashdown’s agar for colony counts to evaluate the MBCs. The MBC must have decreased 99.9% of the bacterial cell count when compared to growth control.

### Statistical data analysis

All experiments were performed as three independent replicates and expressed as mean ± standard deviation. Data were subjected to analysis of variance using IBM SPSS software (SPSS Inc., Chicago, IL, USA).

## Result

### Production time of antibacterial compound

**Fig. 1:**
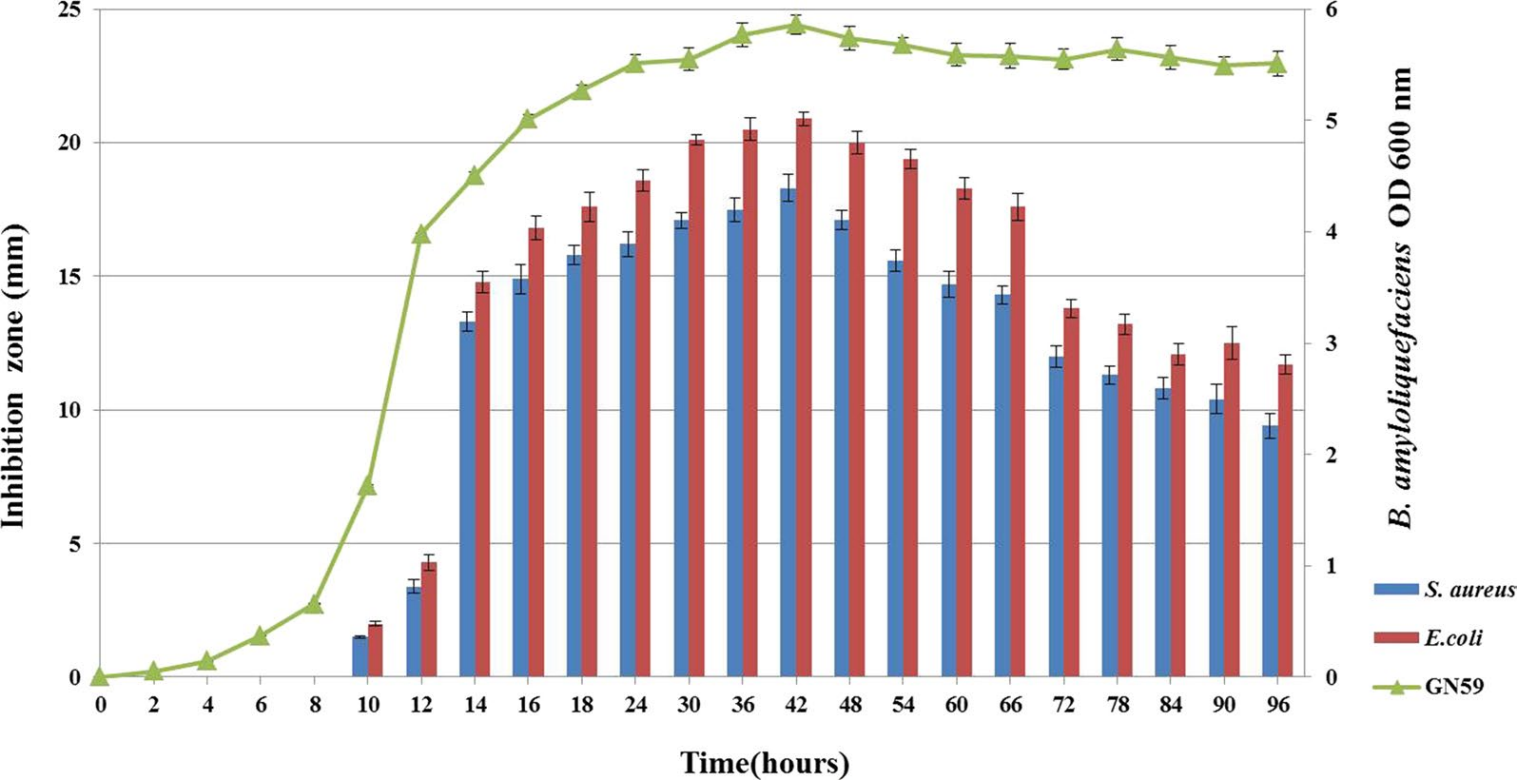
The production of antibacterial compound from *B. amyloliquefaciens* GN59. The production of secondary metabolites displayed as sizes of the inhibition zones against *S. aureus* (blue bars) and *E. coli* (red bars) by the agar well diffusion method on the left Y axis and growth curve as measured at OD600 nm of GN59 was plotted on the right Y axis. X axis represented the time in hours

The metabolites from GN59 showed a broadened antimicrobial activity. After being cultured for 12 h, the culture supernatant started to show antibacterial activity against *S. aureus* ATCC6538 and *E. coli* CMCC44102. The highest activity was observed at 42 h with the inhibition zone of 18.3 and 20.9 mm separately (Fig. 1).

### Purification of the antibacterial compound

**Fig. 2:**
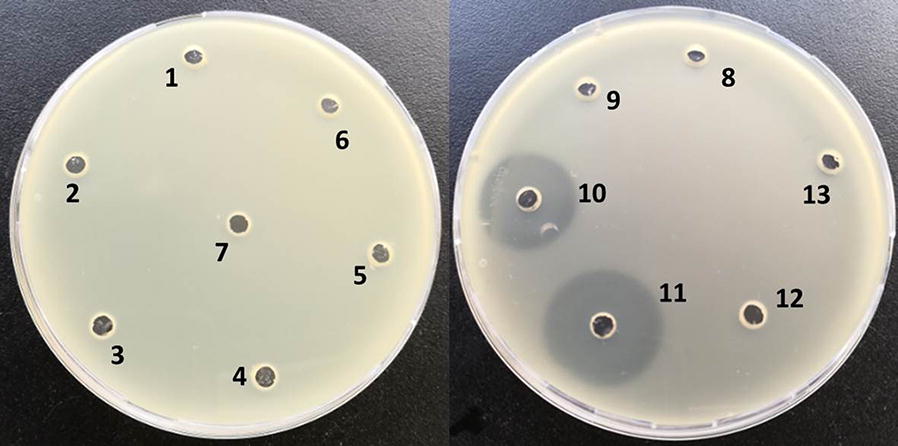
The antibacterial activity of the elution gradient of silica gel column chromatography. Antimicrobial activity of five fractions of ethyl acetate phase of GN59 fermentation broth the number 1, 2, 3, 4, 5 represents different fractions eluted by eluents (petroleum ether:ethyl acetate 100:0; petroleum ether:ethyl acetate 75:25; petroleum ether:ethyl acetate 50:50; petroleum ether:ethyl acetate 25:75; petroleum ether:ethyl acetate 0:100). 6 and 7 represent fractions eluted by ethyl acetate: methanol 75:25. 8 and 9 represent fractions eluted by ethyl acetate: methanol 50:50; 10 and 11 represent fractions eluted by ethyl acetate: methanol 25:75. 12 and 13 represent fractions eluted by ethyl acetate: methanol 0:100. The No. 10 and 11 have antibacterial activity agaist *E.coli*. The experiment was repeated three times

**Fig. 3:**
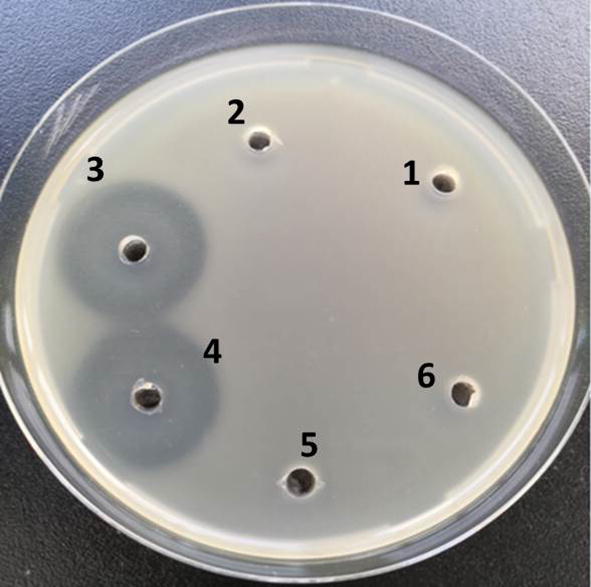
Antibacterial activity of HPLC collected samples. 1, 2, 3, 4, 5, 6 respectively represent the received samples from period 0.0–1.0 min, 1.0–5.1 min, 5.1–5.5 min, 5.1–5.5 min, 5.5–6.5 min, 6.5–7 min. No. 3 and 4 is both from 5.1–5.5 min which have antibacterial activity against *E.coli*. The experiment was repeated three times

The antibacterial effects of the GN59 were evaluated against *E. coli*. 2 L of specific fermentation medium in a 5 L flask was inoculated at 37 °C, 180 rpm for 42 h. An antibacterial compound was purified from the fermentation supernatant of GN59 by bioactivity guided purification. The fraction eluted from eluent (ethyl acetate: methanol 25:75) showed high antibacterial activity. Other fractions didn’t have an antibacterial effect (Fig. 2). The active component (0.5 g) was dissolved in 10.0 mL of methanol and further purified by HPLC. Fraction A (0.0–1.0 min), fraction B (1.0–5.1 min), fraction C (5.1–5.5 min), fraction D (5.5–6.5 min) and fraction E (6.5–7 min) were collected at different period. The results showed that fraction C had antibacterial activity against *E. coli* (Fig. 3).

### Structural analysis of antibacterial compound

**Fig. 4:**
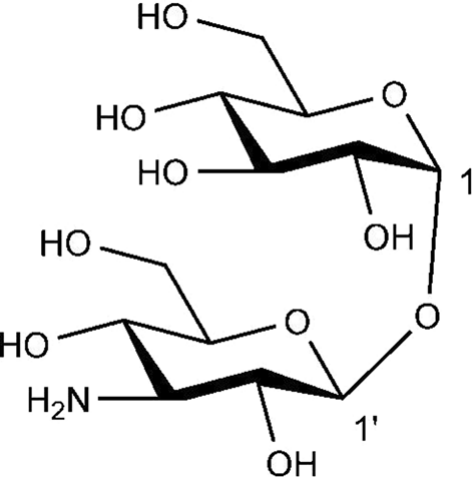
Structures of 3′-neotrehalosamine. The antibacterial compound was 3′-neotrehalosamine(α-d-glucopyranosyl-(1 → 1′) -3′-mino-3′-deoxy-β-d-glucopyranoside). Structural formula: C_12_H_23_NO_10_

Assignments of ^1^H NMR, ^13^C NMR and MS of compound C are as follows: ^1^H NMR (CDCl_3_, 400 MHz): δ 4.95 (d, *J *= 2.8 Hz, 1H), 4.37 (d, *J *= 7.7 Hz, 1H), 3.68 (d, *J *= 10.0, Hz, 1H), 3.66–3.61 (m, 2H), 3.57–3.53 (m, 4H), 3.40–3.20 (m, 4H), 2.83 (t, *J*¼ 8.5 Hz, 1H) (Additional file 1: Fig. S1); ^13^C NMR (CDCl_3_, 100 MHz): δ 97.1 (C-1), 92.5 (C-1′), 76.9 (C-5), 76.9 (C-5), 75.1 (C-3), 73.3 (C-5′), 72.6 (C-3′), 72.1 (C-4), 0.8 (C-4′), 70.5 (C-2), 61.4 (C-6), 61.1 (C-6′) (Additional file 1: Fig. S2); HRMS (positive) *m/z* 342.1402 [M + H]^+^ (Additional file 1: Fig. S3). The chemical formula of the compound is C_12_H_23_NO_10_ after analysis (Fig. 4) which is the same with the substance 34. (Shazia et al. 2013).

The results revealed that the antibacterial compound of GN59 was proposed to be α-d-glucopyranosyl-(1 → 1′)-3′-amino-3′-deoxy-β-d-glucopyranoside (a,β-3-trehalosamine). It is identified as a new trehalosamine, which has the potential to be safe and environmentally friendly bactericide. This is the first report on antibacterial activity of a new trehalosamine from biological resources. The biologically sourced a,β-3-trehalosamine with antibacterial activity that we discovered represents a new antibacterial compound resource.

### Broad-spectrum antimicrobial activities of antibacterial compound

**Fig. 5:**
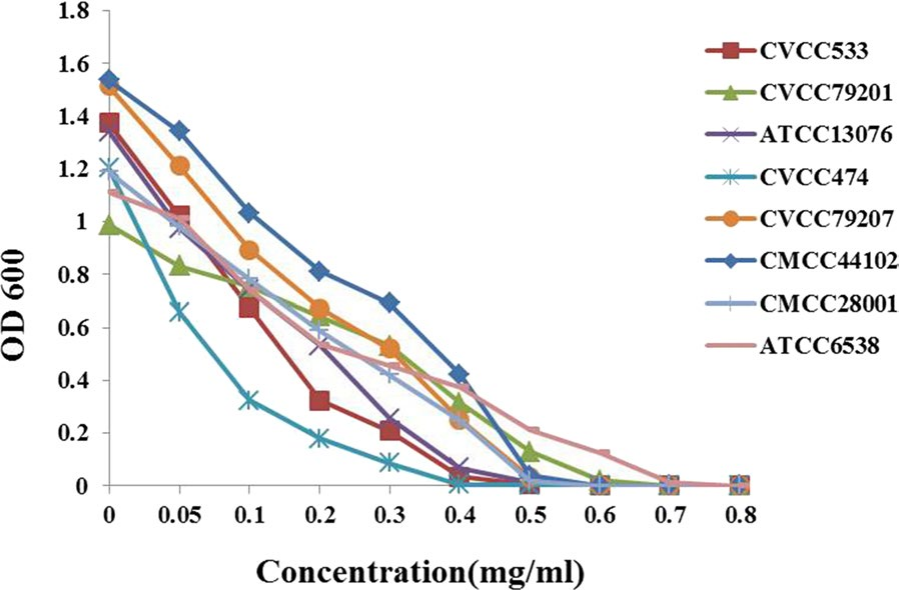
Broad-spectrum antimicrobial activities was investigated. X axis represented the concentration of a,β-3-trehalosamine and growth curve as measured at OD600 nm of GN59 was plotted on the right Y axis

The new compound a,β-3-trehalosamine was determined by the well-diffusion to test the broad-spectrum antimicrobial activity and it had a broad-spectrum activity against different gram-positive and gram-negative organisms including *S. aureus* ATCC6538, *M. luteus* CMCC280010, *E. coli* CMCC44102, *S. pullorum* CVCC533, C79-13, *S. gallinarum* CVCC79201, C79-1, *S. gallinarum* CVCC79207, C79-7, *P. multocida* CVCC474, C48-7 and *S. enteritidis* ATCC13076 (Table 1). The minimum inhibitory concentration (MIC) of a,β-3-trehalosamin was 0.7, 0.6, 0.5, 0.5, 0.6, 0.6, 0.4 and 0.5 mg/mL, respectively (Fig. 5).

**Table 1:** Diameters (mm) of the zones of inhibition of growth of all probed bacteria

Bacteria	Diameters (mm) of the zones (0.75 mg/mL)
*Staphylococcus aureus* (ATCC6538)	8 ± 1
*Micrococcus luteus* (CMCC280010)	12 ± 1
*Escherichia coli* (CMCC44102)	14 ± 1
*Salmonella pullorum* (CVCC533, C79-13)	15 ± 1
*Salmonella * variant *Salmonella gallinarum* (CVCC79201, C79-1)	11 ± 2
*Salmonella gallinarum* (CVCC79207, C79-7)	11 ± 1
*Pasteurella multocida* (CVCC474, C48-7)	17 ± 1
*Salmonella enteritidis* (ATCC13076)	14 ± 1

### Time‑kill assay of antibacterial compound (MBC)

**Fig. 6:**
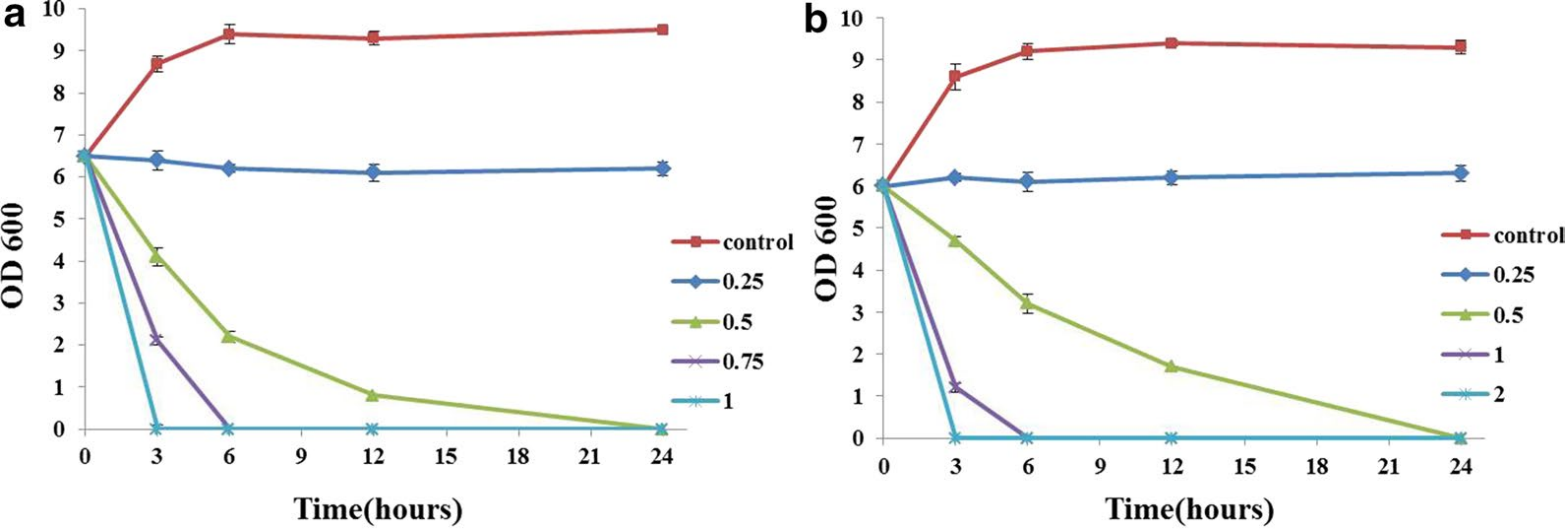
Time-kill assay of active component from GN59 against *E. coli* (**a**) and *S. aureus* (**b**). The X axis indicates time in hours after various concentrations of the trehalosamine as indicated (mg/mL) were added into *E. coli* (**a**) and *S. aureus* (**b**) cultures and the Y axis indicates the CFU/mL of *E. coli* (**a**) and *S. aureus* (**b**) after treatment

The results from MBC assay showed that 3′-neotrehalosamine from GN59 could kill *E. coli* and *S. aureus*. *E. coli* could be killed by 3′-neotrehalosamine at concentrations of 0.5, 0.75 and 1.0 mg/mL within 24, 6 and 3 h separately (Fig. 6a). *S. aureus* could be killed by 3′-neotrehalosamine at concentrations of 0.5, 1.0 and 2.0 mg/mL within 24, 6 and 3 h separately (Fig. 6b). The killing activity of the active compound was in a dose dependent manner.

## Discussion

*Bacillus* spp. have been utilized extensively as biopesticides and fungicides in animal feed (Zhao et al. 2017; Mingmongkolchai et al 2017, 2018). *B. amyloliquefacien* is a non-pathogenic aerobic gram-positive bacterium and widely exists in nature. In this study, we report the purification, structure elucidation and antimicrobial activity of α-d-glucopyranosyl-(1 → 1′)-3′-amino-3′-deoxy-β-d-glucopyranoside (a,β-3-trehalosamine)) from *B. amyloliquefaciens* GN59 fermentation broth. This component displayed strongly antimicrobial activity against most of the pathogenic bacterium. This is the first report on antibacterial activity of a new trehalosamine from biological resources. The biologically sourced trehalosamine with antibacterial activity that we discovered represents a new antibacterial component resource. It showed that the metabolites of probiotics are a potential source for the discovery of new beneficial substances. In addition, the a,β-3-trehalosamine from GN59 has the potential to be safe and environmentally friendly. Therefore, it may facilitate the potential application in the livestock and poultry farming.

Studies have found that a number of metabolites from microbial strains including lipopeptide, macrolide, surfactin (Santos et al. 2018), fengycin (Fan et al. 2017), iturin (Zhang et al. 2017), bacillomycin D (Tabbene et al. 2016), chitinase (Shehata et al. 2018), and other active proteins (Li et al. 2009) have antimicrobial activity. It is reported that the mainly antimicrobial substances produced by *Bacillus* are presented with different proteins and lipopeptides (Abriouel et al. 2011). The reported non-protein antimicrobial substances mainly include macrocyclic lipids, polyene, phenols (Pinheiro et al. 2018), fatty alcohols (Hayama et al. 2015). However, the antibiotic activity of trehalosamine from bacterium in our study has not been previously reported. a,β-3-trehalosamine has a broad-spectrum antimicrobial activities including different gram-positive and gram-negative bacteria. As far as two gram-positive and five gram-negative strains were tested, the antibacterial activity against gram-negative bacteria was stronger than against gram-positive ones. It may be related to the different membrane structure of pathogenic bacteria. We calculate that the antimicrobial effect mechanism of a,β-3-trehalosamine may destroy membrane structure pathogenic bacteria and cause the death of the pathogenic bacteria. But it needs to be further investigated. The properties of the component suggest a path towards developing biological antibiotic component that are likely to avoid development of multidrug resistance.

During the metabolic process, *B. amyloliquefaciens* produces a number of bioactive substances which have antibacterial activity, immunocompetence, antioxidant activity. So it is generally applied in animal husbandry and aquaculture. At the same time, it is expected to provide information for further exploration and application. The a,β-3-trehalosamine we found has strong activity and is easy to synthesize (Shazia et al. 2013). What’s more, as a natural antimicrobial substances synthesized by some microorganisms, a,β-3-trehalosamine show great potential as antibiotics alternatives.

## Supplementary information

**Additional file 1. Fig S1.** Positive HR-ESI-MS data for active substance. **Fig S2.** 1H NMR spectrum active subsatnce in D_2O_.** Fig S3.** ^13^C NMR spectrum of active substance in D_2O_.

## Data Availability

All data obtained have been included into the manuscript.
