# Supplementary material for: A novel trehalosamine isolated from Bacillus amyloliquefaciens and its antibacterial activities
Source: AMB Express. 2020 Jan 14;10:6. doi: 10.1186/s13568-019-0943-x (PMC6960277; doi:10.1186/s13568-019-0943-x)
Supplement: Supplementary file 1 — Additional file 1. Fig S1. Positive HR-ESI-MS data for active substance. Fig S2. 1H NMR spectrum active subsatnce in D2O. Fig S3. 13C NMR spectrum of active substance in D2O. [file 13568_2019_943_MOESM1_ESM.pdf]

Supplementary Materials:

AMB Express

A Novel trehalosamine isolated from *Bacillus amyloliquefaciens* and antibacterial activities

Ying Wang[1], Bo Zhao[1], Yaping Liu[2], Linjing Mao[3], Xuanming Zhang[1], Kechun Liu[1], Jie Chu[*1]

[1]Biology Institute, Qilu University of Technology (Shandong Academy of Sciences)，Ji'nan, Shandong, 250103,China

[2]School of Medical Instrument and Food Engineering, University of Shanhai for Science and Technology, Shanghai, 200093, China

[3]College of animal science and technology of Shandong Agricultural University, Tai'an, Shandong, 271018, China

***Correspondence:** Jie Chu, Shandong Academy of Sciences, 28789 East Jingshi Road Ji'nan, Shandong, 250103, China. Email:**chujie6532@163.com**

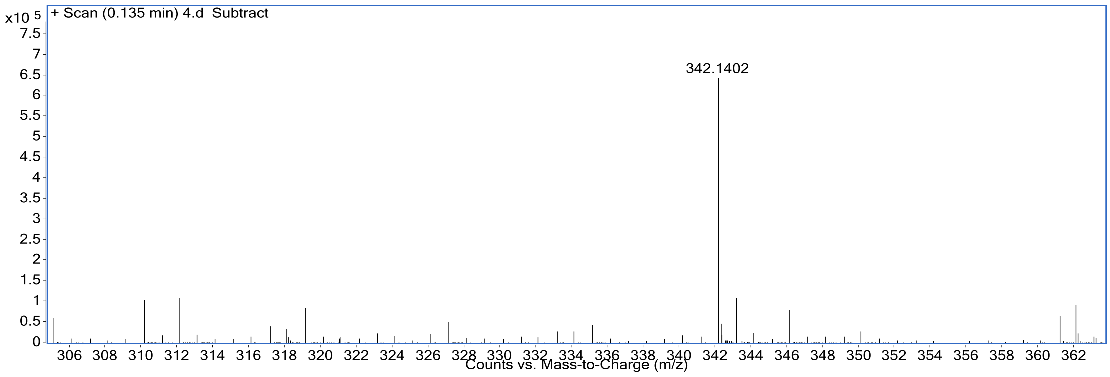

Fig S1: Positive HR-ESI-MS data for active substance.

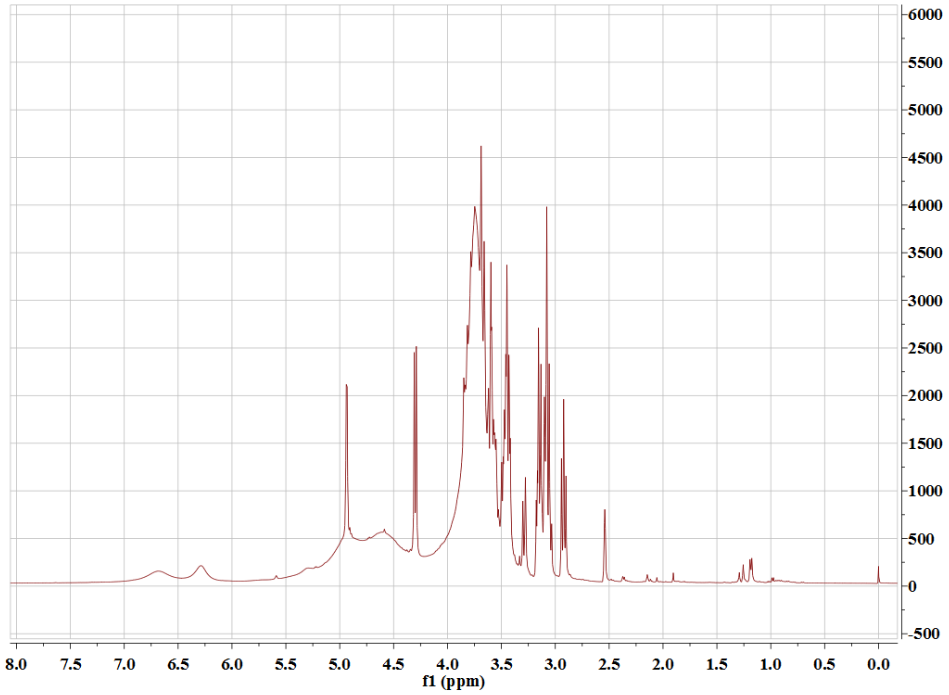

Fig.S2 1H NMR spectrum of active substance in D_2O.

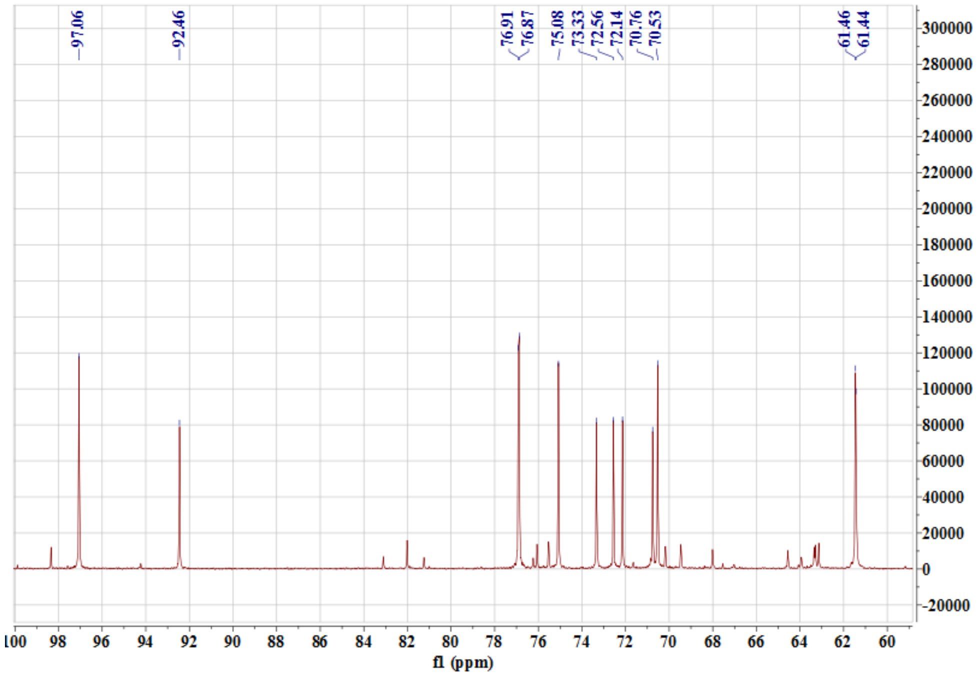

Figure S3 ^{13}C NMR spectrum of active substance in D_2O.
